# Insulin Degludec Versus Insulin Glargine on Glycemic Variability in Diabetic Patients: A Systematic Review and Meta-Analysis of Randomized Controlled Trials

**DOI:** 10.3389/fendo.2022.890090

**Published:** 2022-05-26

**Authors:** Yunjiao Yang, Cong Long, Tongyi Li, Qiu Chen

**Affiliations:** ^1^ Hospital of Chengdu University of Traditional Chinese Medicine, Chengdu, China; ^2^ School of Clinical Medicine, Chengdu University of Traditional Chinese Medicine, Chengdu, China

**Keywords:** glycemic variability, insulin degludec, insulin glargine, diabetic patients, meta-analysis

## Abstract

**Background/Aims:**

Currently, glycemic variability has more deleterious effects than sustained hyperglycemia and is closely associated with acute and chronic complications of diabetes. Reducing glycemic excursion is becoming another vital goal of glycemic control in clinical practice. This study aimed to determine whether insulin degludec (IDeg) or insulin glargine (IGla) was more beneficial for reducing glycemic fluctuations.

**Materials and Methods:**

This research was constructed according to the PRISMA guidelines. We searched eight databases and ClinicalTrials.gov from their inception to 30 November 2021. All randomized controlled trials comparing the efficacy of glucose variability between IDeg and IGla in diabetic patients were included.

**Results:**

Fourteen trials with 8,683 participants were included. In patients with T1DM, IDeg was associated with a lower mean (MD: −16.25, 95% CI −29.02 to −3.07, P = 0.01) and standard deviation (P = 0.03) compared to IGla in fasting blood glucose (FBG); in people with T2DM, IDeg was related to a lower mean of FBG versus insulin glargine 100 U/ml (IGla100) (P <0.001) and had a more extended time in the range (TIR) than IGla100 (SMD: 0.15, 95% CI 0.02 to 0.27, P = 0.02) but not longer than insulin glargine 300 U/ml (IGla300). Moreover, IDeg had a lower coefficient of variation of FBG than IGla (P = 0.0254). For other indicators of glycemic variability, namely, standard deviation of blood glucose for 24 h, the mean of 24-h blood glucose, mean amplitude of glycemic excursion, the coefficient of variation for 24 h, the mean of daily differences, area under the glucose curve, and M-value, no significant differences were identified between IDeg and IGla, regardless of T1DM or T2DM.

**Conclusions:**

Based on the current studies, there was comparable efficacy between IDeg and IGla from multiple aspects of glycemic variability, regardless of T1DM or T2DM. However, IDeg may be superior to IGla in reducing FBG variability in T1DM and T2DM. Nonetheless, due to the limitations of the original studies, it is still unclear whether IDeg is superior to both IGla100 and IGla300. In T2DM, IDeg had more extended TIR than IGla100 but not longer than IGla300. Additionally, more well-designed randomized controlled trials comparing IDeg with IGla300 for different indicators of glycemic variability are still warranted.

**Systematic Review Registration:**

PROSPERO, CRD42021283203.

## Introduction

Diabetes mellitus (DM) refers to a group of metabolic disorders characterized by high blood glucose levels. It was estimated that in 2017, there were 451 million people with diabetes globally, which has been increasing consistently and is expected to increase to 693 million by 2045 (1). In the past, glycated hemoglobin tended to be the reference parameter indicating the risk of complications for treating diabetes mellitus. However, recently, a new important clinical dynamic parameter has emerged for glycemic control. It is glycemic variability (GV), which refers to the unstable state of blood glucose levels changing between trough and peak. Studies have shown that glucose fluctuations have more deleterious effects than sustained hyperglycemia (2–4). Additionally, there was increasing evidence that glucose excursions were associated with a growing risk of diabetic macrovascular and microvascular complications, hypoglycemia, mortality rates, and other adverse clinical outcomes (5–10). Therefore, reducing glycemic instability is becoming another goal of blood glucose control in clinical practice. Insulin use is widespread and necessary for advanced type 2 diabetes mellitus (T2DM) and type 1 diabetes mellitus (T1DM). Consequently, it is vital to choose optimal insulin with a lower risk of glycemic variability.

Insulin glargine (IGla) and insulin degludec (IDeg) are common once-daily basal insulins at present, and they both have been associated with a constant glucose-lowering effect throughout the day (11, 12). IDeg was reported to provide sustained glucose-lowering efficacy for more than 42 h, with its half-life extended to 25 h (13), which is much longer than IGla. However, the time to reach a stable state could be correspondingly longer. In a study comparing the efficacy and safety of insulin glargine 100 U/ml (IGla100) and insulin degludec100 U/ml (IDeg100) in patients with T2DM, the standard deviation (SD) and coefficient of variation (CV) of blood glucose on day 7 were higher in the IDeg100 group, indicative of greater glucose fluctuation (14). Balsells et al. demonstrated that insulin glargine 300 U/ml (IGla300) provided more stable pharmacodynamic and distributed pharmacokinetic characteristics compared with IDeg100 (15). However, other studies have suggested that IDeg has lower day-to-day variability and a more stable glucose-lowering effect than IGla (16–19). Apparently, there was disagreement about whether IDeg or IGla was more effective for glycemic variability. Though meta-analyses showed IDeg has a significantly lower rate of nocturnal and overall hypoglycemia in patients with diabetes (20, 21), there was no study to systematically review the efficacy of glycemic variability between IDeg and IGla. Therefore, this study is expected to provide sufficient and valid evidence.

## Methods

### Study Registration

This study was registered in PROSPERO (CRD42021283203) and was designed in accordance with the guidelines for preferred reporting items for systematic reviews and meta-analyses (PRISMA 2020) (22).

### Databases and Search Strategies

Eight common databases were searched from their inception to 30 November 2021, specifically including the Cochrane Library, PubMed, Embase, Web of Science, Chinese Biomedical Literature Database (CBM), Chinese National Knowledge Infrastructure (CNKI), VIP database, and Wanfang database. Besides, Clinical Trials (ClinicalTrials.gov), unpublished gray literature, and references cited in the eligible studies were also searched. We used a search strategy that combined MeSH terms with free-text words. For searching in PubMed, as an example, the following terms were used: “Insulin glargine”[Mesh]OR ((((Glargine) OR (Lantus)) OR (Glar)) OR (“Recombinant Insulin Glargine Injection”)) OR (“Insulin Glargine Injection”) AND “Insulin degludec”[Mesh] OR ((Tresiba) OR (Degludec)) OR (IDeg) AND (((((((((“glucose fluctuation”) OR (“glucose excursion”)) OR (“glucose variability”)) OR (“glycemic fluctuation”)) OR (“glycemic excursion”)) OR (“glycemic variability”)) OR (“glucose variation”)) OR (“glycemic variation”)) OR (“glucose instability”)) OR (“glycemic instability”).

### Eligibility and Exclusion Criteria

Eligibility criteria: *Patients*: All diabetic patients were included, irrespective of the types of diabetes mellitus; *Intervention/comparison*: IDeg versus IGla for the efficacy of glucose variability with no restriction on the number of cases and the intervention time. If IDeg and IGla were additional treatments, the other treatment must be the same in both groups; *Outcomes*: results of glycemic excursion must contain at least one of the following: standard deviation of blood glucose (SDBG), mean amplitude of glycemic excursions (MAGE), mean blood glucose (MBG), time in the range (TIR), a mean of daily differences (MODD), the coefficient of variation (CV), area under the glucose curve (AUC), and M-value; *Study design*: randomized controlled trials.

Exclusion criteria: animal experiments, retrospective studies, case reports, abstracts, and protocols were excluded.

### Study Selection and Data Extraction

Two researchers (YY and CL) searched and extracted the articles based on inclusion and exclusion criteria, respectively. Conflicts were resolved by a third reviewer (QC). Duplicates were removed by software (Endnote X9). After reading the title, abstract, and full text, eligible trials were selected. The following data were extracted and saved in an Excel form: the first author, publication time, study design, country, type of diabetes, total cases, the number of cases in the IGla group, number of cases in the IDeg group, specific treatment methods for each group, follow-up time, baseline data including age, sex ratio, duration of diabetes, outcomes of glycemic variability. Concerning the crossover-controlled experiment, if the results of the two stages in the paper were reported separately, we extracted the results of the first stage. For articles that lacked enough data or data not available, we contacted the corresponding authors for more details by email. If unsuccessful, we only analyzed the available data.

### Risk of Bias and Quality Appraisal

The risk of bias of included studies was independently assessed by two authors (YY and TL), and disagreements were resolved by the third reviewer (QC). The Cochrane Collaboration tool was used to assess the risk of bias (23). Each item was classified as low, unclear, or high risk of bias for seven specific domains.

### Data Synthesis and Statistical Analysis

Review Manager 5.3 software was used to perform the meta-analysis. The effect indicators were expressed as the mean difference (MD) and 95% confidence interval (95% CI). The standard mean difference (SMD) was used for the same metric with different measurement methods. For the studies reporting values with median and interquartile ranges, the values were converted to mean and standard deviations by appropriate methods (24). For indicators of blood glucose reported in different units (mmol/l; mg/dl) in the included studies, we unified the units to mg/dl. Cochran Q and I^2^ statistics were used to evaluate statistical heterogeneity between studies, and the Q statistics >the df with a p-value of <0.1, which indicates significant heterogeneity. A random-effect model was used to perform the meta-analysis. Considering the diverse influence of different types of diabetes on glycemic fluctuation, we analyzed TIDM and T2DM, respectively. Subgroup analysis was performed according to HbA1c level and the type of IGla. A P-value of less than 0.05 was considered statistically significant. No meta-regression or sensitive analysis was conducted for further analysis. Because of insufficient studies for each metric of glycemic variability, publication bias was not undertaken.

## Results

### Search Results

**Figure 1:**
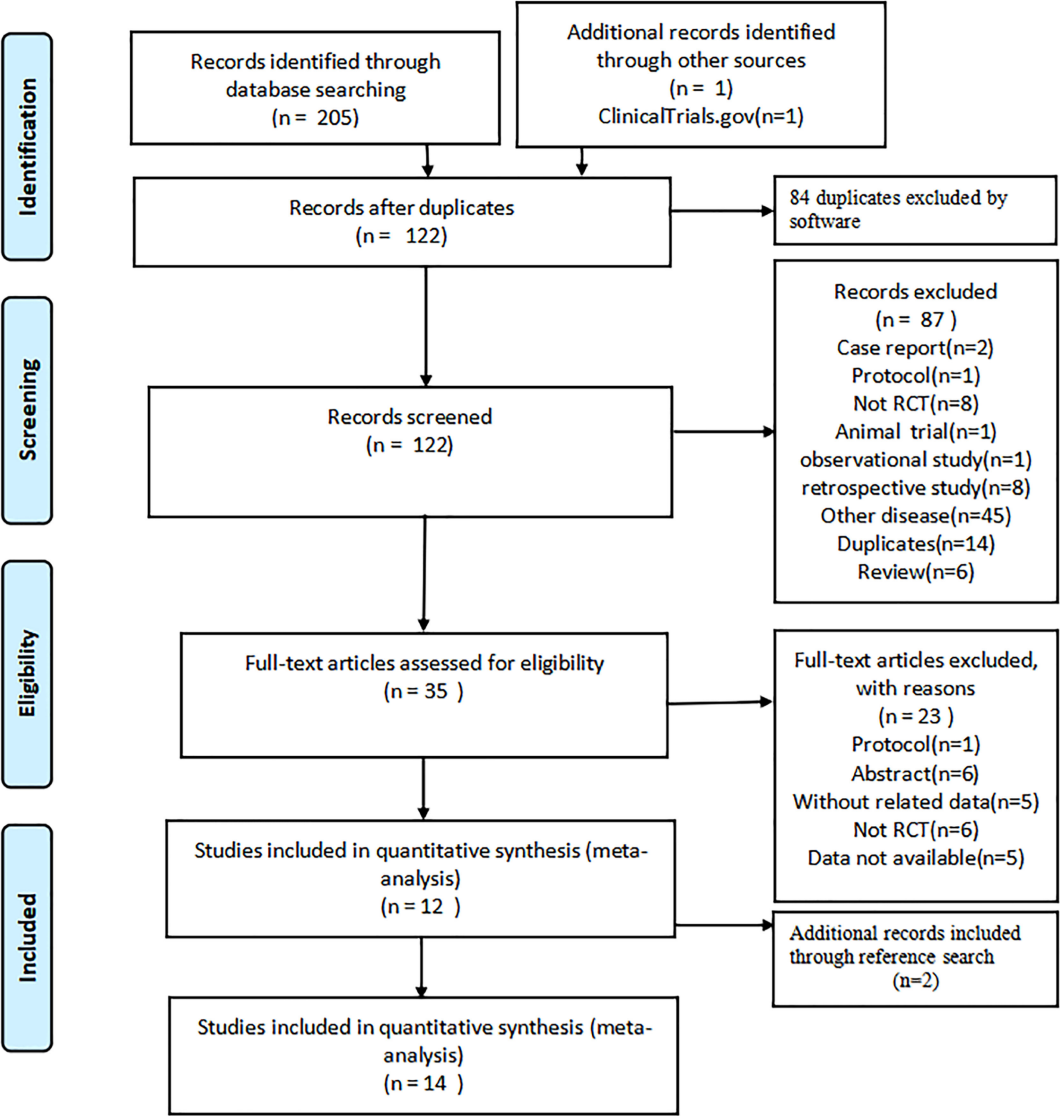
The flowchart of the study selection process.

A total of 206 citations were identified from the following databases: PubMed (n = 8), Web of Science (n = 37), Embase (n = 88), Cochrane library (n = 58), CNKI (n = 2), Wanfang (n = 5), VIP (n = 3), CBM (n = 4), ClinicalTrials.gov (n = 1). No gray literature was found. Eighty-four duplicates were removed by software, and the remaining studies were screened by reading their titles and abstracts; 35 relevant full-texts were selected for further consideration. By reading the full text, 23 studies were excluded: Protocol (n = 1), Abstract (n = 6), Without related data (n = 5), Not RCT (n = 6), and Data not available (n = 5). Then twelve RCTs met the eligibility criteria (13, 14, 17–19, 25–31). Two additional records (32, 33) met the eligibility criteria by reading the references. Finally, 14 studies involving 8,683 patients were included in this research. The flowchart of study selection is presented in **Figure 1**.

### Study Characteristics

Of the 14 articles included, eight were crossover studies (13, 17, 18, 25–28, 32), and the other studies used a parallel design (14, 19, 29–31, 33). Five of the 14 were multicenter studies (17, 19, 26, 27, 33). The duration of follow-up ranged from 6 days to 41 weeks, but three studies did not report the duration of follow-up (29–31). The sample size ranged from 12 to 7,637. Ten studies were conducted in T2DM (14, 19, 25, 26, 28–33) and four were undertaken in T1DM (13, 17, 18, 27).The trials were conducted in the following regions: Japan (n = 8), China (n = 3), Mexico (n = 1), Canada (n = 1), and America (n=1). The publication year ranged from 2015 to 2021. There were four studies in which the treatment regimen was insulin aspart combined with IDeg and IGla. Five of the studies used insulin glargine 300 U/ml (IGla300) (27–30, 32); three articles used insulin glargine 100 U/ml (IGla100) (14, 26, 33); other studies did not specify what type of IGla was used (13, 17–19, 25, 31). The mean age of patients ranged from 44.1 to 71.9 years. The mean duration of diabetes ranged from 4.12 to 19.4 years, and the mean HbA1c level at baseline ranged from 6.78 to 11.3%. The specific baseline characteristics of the eligible studies are shown in **Table 1**.

**Table 1:** Baseline characteristics of included studies.

First author and year	Design	Country	Follow-up	Patients	Male (%)	Total cases	Sample size		Treatment		Age (years)		Disease duration (years)		HbA1c (%)		Outcomes
							IDeg	IGla	Group1	Group2	IDeg	IGla	IDeg	IGla	IDeg	IGla	
Yoshiko, 2016 (13)	RCT, C	Japan	8 weeks	T1DM	54%	13	13^☆^	13^☆^	IDeg^★^	IGla^▲^	44.9 (7.2)	44.9 (7.2)	15.5 (7.0)	15.5 (7.0)	7.8 (0.54)	7.9 (0.54)	①②③④
RyoIga, 2017 (18)	RCT,O,C	Japan	24 weeks	T1DM	55%	20	10	10	IAsp + IDeg^★^	IAsp + IGla^▲^	54 (16)	54 (16)	16 (8)	16 (8)	7.1 (0.9)	7.7 (0.6)	②⑤⑦
Yuji, 2019 (28)	RCT,O,C	Japan	10 days	T2DM	60%	30	15	15	IDeg^★^	IGla300	69.5 (11.3)	69.5 (11.3)	18.3 (11.3)	18.3 (11.3)	8.0 (1.5)	8.5 (2.2)	①②③⑤⑥⑦⑧
Tomoaki, 2015 (17)	RCT,O,M,C	Japan	8 weeks	T1DM	41%	36	17	19	IDeg^★^	IGla^▲^	57 (14)	57 (14)	18 (10)	18 (10)	7.4 (0.8)	7.4 (0.8)	①②⑥
Yoshimasa, 2017 (19)	RCT,O,M,P	Japan	24 weeks	T2DM	45%	43	31	12	IDeg^★^	IGla^▲^	64.0 (13.6)	64.7 (15.7)	10 (3.5)	14.5 (5.27)	8.88 (1.48)	8.84 (1.46)	⑥
Hiroshi, 2020 (27)	RCT,M,C	Japan	4 weeks	T1DM	30%	46	23	23	IDeg^★^	IGla300	53.3 (14.7)	53.3 (14.7)	19.4 (11.6)	19.4 (11.6)	7.6 (0.7)	7.6 (0.7)	①②③⑤⑥⑦⑧
Jun, 2019 (14)	RCT,O,P	Japan	12 days	T2DM	51%	74	36	38	IDeg100	IGla100	58.9 (10.5)	61.8 (9.4)	3.9 (4.6)	6.6 (8.2)	11.3 (1.4)	10.4 (1.9)	①②③⑤⑥
Yan.Han, 2020 (31)	RCT,P	China	NR	T2DM	58%	64	32	32	IAsp + IDeg^★^	IAsp + IGla^▲^	52.38 (6.29)	52.54 (6.07)	10.34 (1.25)	10.29 (1.54)	9.12 (1.46)	9.07 (1.34)	②⑤
LiTian, 2019 (30)	RCT,P	China	NR	T2DM	67%	86	43	43	IAsp+IDeg300	IAsp + IGla300	53.3 (8.8)	53.9 (8.5)	NR	NR	11.2 (1.8)	11.4 (1.7)	⑤
Qing, 2020 (29)	RCT,P	China	NR	T2DM	59%	100	30	70	IAsp + IDeg300	IAsp + IGla300	57.96 (8.35)	58.74 (8.41)	4.23 (1.05)	4.12 (1.03)	11.29 (1.74)	11.25 (1.85)	⑤
Ronald, 2021 (26)	RCT,O,M,C	Canada	41 weeks	T2DM	48%	498	249	249	IDeg100	IGla100	62.9 (10.0)	62.7 (9.7)	14.5 (7.0)	15.6 (8.3)	7.6 (1.0)	7.6 (1.0)	⑤
Nct, 2020 (25)	RCT,C	Mexico	6 days	T2DM	67%	12	6	6	IDeg^★^	IGla^▲^	44.1 (8.8)	44.1 (8.8)	NR	NR	8.2 (1.4)	8.2 (1.4)	③④
Steven, 2017 (33)	RCT,M,D,P	America	2 years	T2DM	63%	7637	3818	3819	IDeg^★^	IGla100	64.9 (7.3)	65.0 (7.5)	16.6 (8.8)	16.2 (8.9)	8.4 (1.6)	8.4 (1.7)	②
Mizuho, 2019 (32)	RCT,O,C	Japan	8 weeks	T2DM	50%	24	12	12	IDeg^★^	IGla300	71.9 (5.2)	69.5 (9.5)	16.5 (9.1)	11.6 (9.1)	6.83 (0.34)	6.78 (0.33)	①②⑤⑥⑦

Data are shown as numbers or means (standard deviation) unless otherwise stated.

^☆^The article did not report sample size of each group. Because it was a crossover study, all participants completed the experiment. So the values in each group are the total sample size; ^★^These studies did not report the type of insulin degludec;^▲^These studies did not report the type of insulin glargine; NR, not report; RCT, randomized controlled trial; O, open-label; M, multicenter; C, crossover; P, parallel; D, double-blind; T1DM, type 1 diabetes mellitus; T2DM, type 2 diabetes mellitus; IDeg, insulin degludec; IGla, insulin glargine; IAsp, insulin aspart; IGla300, insulin glargine 300 U/ml; IDeg100, insulin degludec 100 U/ml; IDeg300, insulin degludec 300U/ml; IGla100, insulin glargine 100 U/ml; HbA1c, hemoglobin A1c; ①, SDBG (standard deviation of blood glucose ); ②, MBG (mean blood glucose); ③, MAGE (mean amplitude of glycemic excursion); ④, AUC (area under the curve of glucose); ⑤, TIR (time in range); ⑥, CV (coefficient of variation); ⑦, MODD (mean of daily difference); ⑧, M-value.

### Risk of Bias in Included Studies

**Figure 2:**
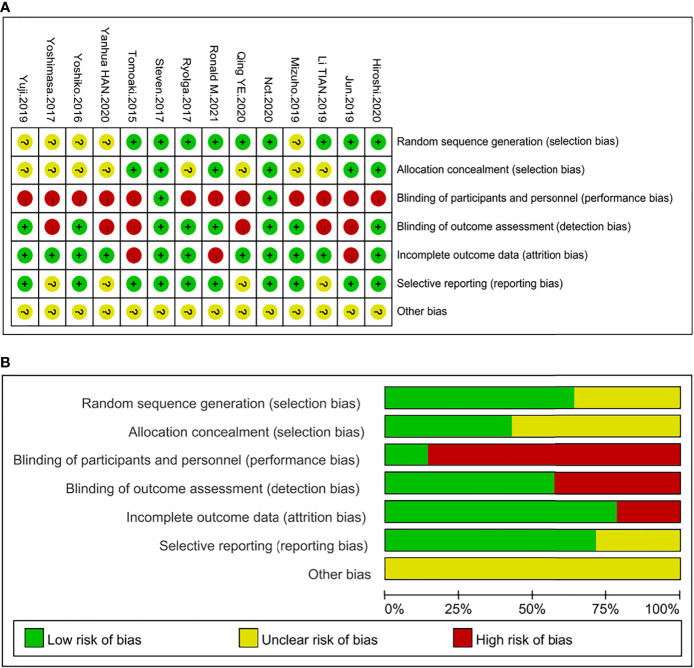
Summary of quality evaluation based on the Cochrane’s Risk of Bias Tool. **(A)** Risk of bias summary for each risk of bias item for each included study; **(B)** Risk of bias graph for each risk of bias item presented as percentages across all included studies.

Among the 14 studies, nine trials described their specific randomization strategies (14, 17, 18, 25–27, 29, 30, 33) and six of them stated the allocation concealment (14, 17, 25–27, 33), which were determined as “low risk” for selection bias. Two studies (25, 33) used the double-blind method and were deemed “low risk” for performance and detection bias. Although six studies (13, 18, 26–28, 32) did not use blinding methods, study results were not affected by using continuous glucose monitoring (CGM) or flash glucose monitoring, which were considered “low risk” for detection bias. As to reporting bias, four trials (19, 29–31) were identified as “unclear risk” because of the failure to register the clinical trial protocol or published protocol before the trial started, and the other nine articles were marked as “low risk.” Regarding attrition bias, three studies (14, 17, 26) were determined as “high risk” because they had withdrawn cases, and the assessment of results could be affected. One study (33) in which a few patients were lost to follow-up and withdrew but not enough to affect overall outcomes was defined as “low risk” along with other articles. All trials were identified as “unclear risk” in terms of other bias. The graph and summary of the risks of bias are shown in **Figure 2**.

### Effect of Standard Deviation of Blood Glucose (SDBG)

**Figure 3:**
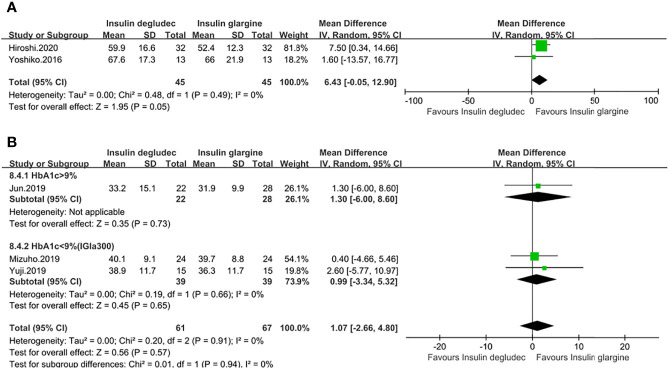
Forest plot for the SD of 24 h**(A)** SD of 24h in patients with type 1 diabetes; **(B)** SD of 24 h in patients with type 2 diabetes. .

SDBG of 24 h was measured in five studies (13, 14, 27, 28, 32). Two studies were conducted on patients with T1DM. One study (27) used IGla300 while the other did not specify the type of IGla (13). Patients in these two studies both had a baseline HbA1c of <9%. The pooled result did not show a significant difference between the two treatment groups (MD: 6.43, 95% CI −0.05 to 12.90, P = 0.05), with no heterogeneity (P_he_ = 0.49, I^2^ = 0%) (**Figure 3A**). Three studies were conducted on patients with T2DM. There were also no significant differences in pooled results between IDeg and IGla (MD: 1.07, 95% CI −2.66 to 4.80, P = 0.57, I^2^ = 0%), and no heterogeneity was identified (P_he_ = 0.91, I^2^ = 0%). Subgroup analysis in two studies (28, 32) using IGla300 with a baseline HbA1c of <9% and in one study (14) using IGla100 with a baseline HbA1c of >9% showed the same outcomes (**Figure 3B** **)**. Only one trial (17) that did not specify the type of IDeg and IGla used in enrolling patients with T1DM reported the result of the standard deviation of fasting blood glucose (SD of FBG), which was lower in the IDeg group than in IGla treatment (P = 0.03).

### Effect of Mean Blood Glucose (MBG)

**Figure 4:**
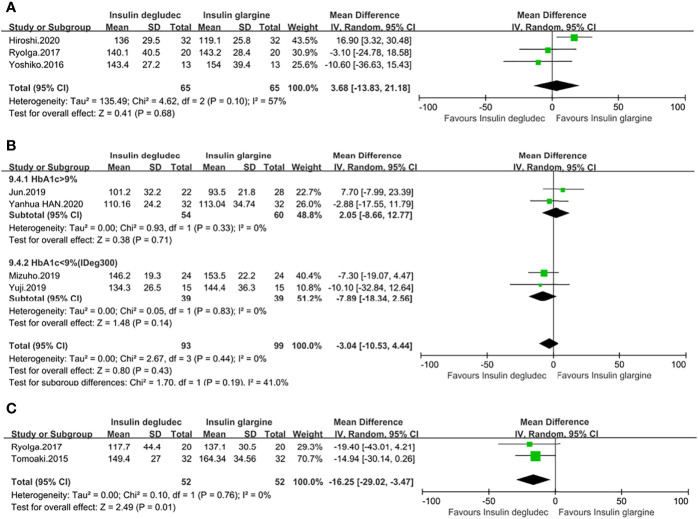
Forest plot for the MBG. **(A)** MBG of 24 h in patients with type 1 diabetes; **(B)** MBG of 24 h in patients with type 2 diabetes; **(C)** The mean of FBG in patients with type 1 diabetes.

MBG of 24 h was reported in seven studies (13, 14, 18, 27, 28, 31, 32). Three studies (13, 18, 27) were undertaken in T1DM patients with a baseline HbA1c of <9%. One study (27) used IGla300, while the other two studies (13, 18) did not report the type of IGla they used. The overall result was similar between the two interventions (MD: 3.68, 95% CI −13.83 to 21.18, P = 0.68), showing no significant heterogeneity (P_he_ = 0.10, I^2^= 57%) (**Figure 4A**). Four studies (14, 28, 31, 32) were conducted in patients with T2DM. Similarly, no significant differences were found in the pooled results (MD: −3.04, 95% CI −10.53 to 4.44, P = 0.43**)**, with no heterogeneity (P_he_ = 0.44, I^2^ = 0%). Two studies (28, 32) with an HbA1c of <9% comparing IDeg with IGla300 showed no difference. Two studies (14, 31) with an HbA1c of >9%, in which one was compared with IGla100 while the other was unknown for the type of IGla, also demonstrated no difference between the two treatment groups. No heterogeneity was found in two subgroup analyses (**Figure 4B**). However, in the case of the mean of FBG, the meta-analysis of two trials (17, 18) with a baseline HbA1c of <9% in T1DM showed that IDeg was more effective than IGla, which was not unknown for the type (MD: −16.25, 95% CI −29.02 to −3.47, P = 0.01), with no heterogeneity (p_he_ = 0.76, I^2^ = 0%) **(** **Figure 4C**). One study (33) enrolling 7,637 patients with a baseline HbA1c of <9% comparing IDeg with IGla100 in T2DM also showed IDeg was more beneficial than IGla100 (P <0.001).

### Effect of Mean Amplitude of Glycemic Excursion (MAGE)

**Figure 5:**
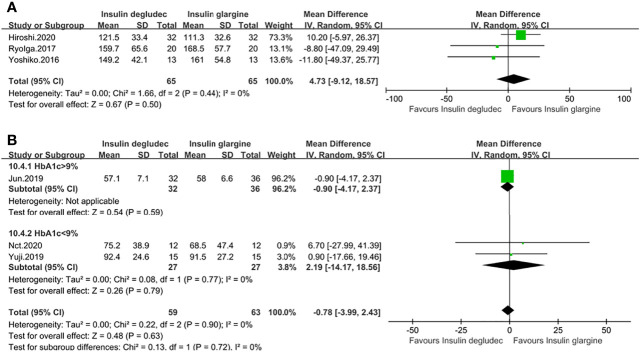
Forest plot for the MAGE. **(A)** MAGE in patients with type 1 diabetes; **(B)** MAGE in patients with type 2 diabetes.

MAGE was reported in six studies (13, 14, 18, 25, 27, 28). Three studies were conducted on patients with T1DM, and their baseline HbA1c levels were all under 9%. One (27) was compared to IGla300, while two (13, 18) did not specify the type of IGla. The pooled result demonstrated no difference between IDeg and IGla (MD: 4.73, 95% CI −9.12 to 18.57, P = 0.50), with no heterogeneity (P_he_ = 0.44, I^2^ = 0%) (**Figure 5A**). Three studies (14, 25, 28) were conducted on participants with T2DM. Pooled results showed no difference between the two treatment groups (MD: −0.78, 95% CI −3.99 to 2.43, P = 0.63), with no heterogeneity (p_he_ = 0.90, I^2^ = 0%). One study (14) had a baseline HbA1c of >9% comparing IDeg with IGla100 showed no discrepancy. A meta-analysis of two trials (25, 28) with a baseline HbA1c of <9%, in which one was compared to IGla300 while the other did not specify the type of IGla, also showed no difference and no heterogeneity between the two treatment groups **(** **Figure 5B**).

### Effect of Time in Range (TIR)

**Figure 6:**
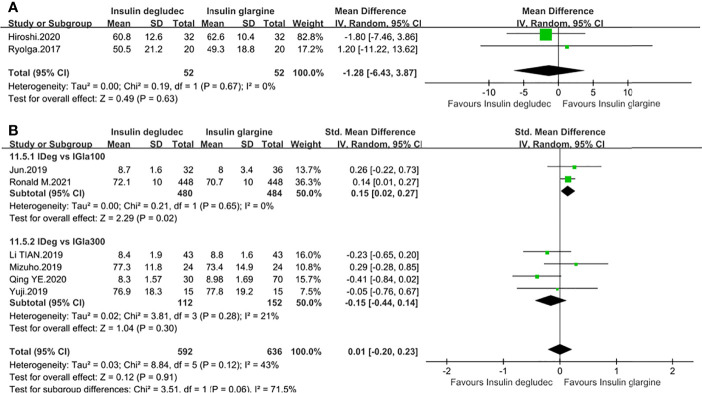
Forest plot for the TIR. **(A)** TIR in patients with type 1 diabetes; **(B)** TIR in patients with type 2 diabetes according to the type of IGla.

Nine trials described TIR change (14, 18, 26–32). Two studies with a baseline HbA1c of <9% were undertaken in T1DM. One (27) was compared to IGla300, and the other (18) was unknown for the type of IGla. No difference was observed in the pooled result (MD: −1.28, 95% CI −6.43 to 3.87, P = 0.63) and no heterogeneity was identified (P_he_ = 0.67, I^2^ = 0%) (**Figure 6A**). Seven studies (14, 26, 28–32) were conducted in T2DM. Two studies (14, 26) comparing IDeg with IGla100 demonstrated that IDeg maintained TIR longer than IGla100 (SMD: 0.15, 95% CI 0.02 to 0.27, P = 0.02) while four studies (28–30, 32) comparing IDeg with IGla300 revealed a comparable effect (SMD: −0.15, 95% CI −0.44 to 0.14, P = 0.30). No significant heterogeneity was observed in two subgroup analyses (**Figure 6B**). Another study (31), without specifying the type of IGla, also showed no difference in TIR compared to IDeg (P >0.05).

### Effect of Coefficient of Variation (CV)

**Figure 7:**
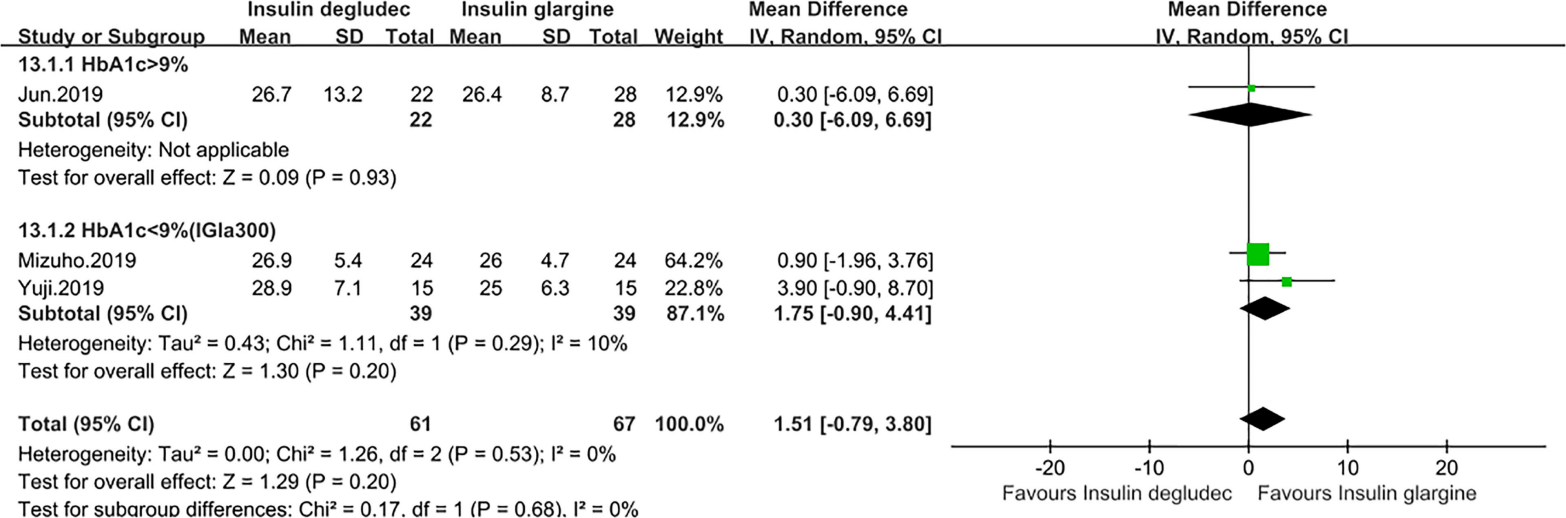
Forest plot for the CV of 24 h in patients with type 2 diabetes.

The CV of 24-h blood glucose was reported in four studies (14, 27, 28, 32). One trial (27) was conducted in patients with T1DM in which the baseline HbA1C level of participants was under 9%. The result identified that IDeg was non-inferior to IGlarU300 (P = 0.68). Three studies were conducted in T2DM, among which two studies (28, 32) with a baseline HbA1c of <9% comparing IDeg with IGla300 and one trial (14), with a baseline HbA1c of >9% comparing IDeg with IGla100, all showed the same efficacy between the two treatments. The pooled result also revealed a comparable effect (MD: 1.51, 95% CI −0.79 to 3.80, P = 0.20) and no heterogeneity (p_he_ = 0.53, I^2^ = 0%) (**Figure 7**). Of note, two trials that did not report the type of IGla and in which the patients all had a baseline HbA1c of <9% assessed the efficacy of CV of FBG. One trial (17) conducted in T1DM showed that IDeg did not differ from IGla (P = 0.32). However, another study (19) made in T2DM demonstrated that the CV of FBG was significantly smaller in the IDeg group than in the IGla group (P = 0.0254).

### Effect of Mean of Daily Differences (MODD)

**Figure 8:**
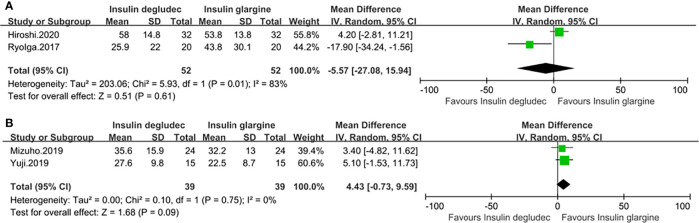
Forest plot for the MODD. **(A)** MODD in patients with type 1 diabetes; **(B)** MODD in patients with type 2 diabetes.

Four studies (18, 27, 28, 32) with a baseline HbA1c of <9% provided the value of MODD. Two studies were conducted in patients with T1DM, in which one study (27) used IGla300 while the other (18) did not specify the type of IGla. The pooled result did not show a significant difference (MD: −5.57, 95% CI −27.08 to 15.94, P = 0.61) but accompanied by great heterogeneity (p_he_ = 0.01, I^2^ = 83%) (**Figure 8A** **)**. Two studies (28, 32) comparing IDeg with IGla300 were conducted in participants with T2DM, and the pooled result revealed that there was also no difference between the two groups (MD: 4.43, 95% CI −0.73 to 9.59, P = 0.09) and no heterogeneity (p_he_ = 0.75, I^2^ = 0%) (**Figure 8B**).

### Effect of Area Under the Glucose Curve (AUC)

Two studies with a baseline HbA1c of <9% (13, 25) reported the change in AUC, but neither of them reported what type of IGla they used. One study (13) undertaken in patients with T1DM demonstrated a comparable effect between IDeg and IGla (P = 0.257). Another study (25) conducted on those with T2DM showed the same result (P = 1.0).

### Effect of M-Value

Two studies with a baseline HbA1C of <9% measured M-value (27, 28), and both of them compared IDeg to IGla300. No significant difference was noted in one study (27) conducted in patients with T1DM (P = 0.10). Another study (28) conducted on those with T2DM also showed no difference between the two treatment groups (P = 0.938).

## Discussion

Over the past decade, glucose variability characterized by both amplitude and timing has been increasingly regarded as a primary marker of glycemic control (34–37). This study determined whether IDeg or IGla is more beneficial for reducing glycemic excursions. Through the analysis of glucose fluctuations from different aspects based on the 14 RCTs, the findings are as follows: In people with T1DM, IDeg was related to a lower mean and SD in FBG compared to IGla. There was comparable efficacy between IDeg and IGla in MAGE, SDBG of 24 h, TIR, MBG of 24 h, CV, MODD, AUC, and M-value. In patients with T2DM, IDeg was associated with a lower mean of FBG versus IGla100. Concerning the CV of FBG, IDeg was also more stable than IGla. Moreover, IDeg achieved TIR longer than IGla100. However, compared with IGla300, IDeg showed similar efficacy in TIR. In terms of MAGE, SDBG, mean of 24 h, CV of 24 h, MODD, AUC, and M-value, there was comparable efficacy between IDeg and IGla.

IDeg100 and IDeg200 were reported to be bioequivalent and had similar pharmacodynamic profiles (38). IGla300 and IDeg are second-generation basal insulin (BI) analogs with improved pharmacokinetic (PK) and pharmacodynamic (PD) properties and a longer duration of action compared with the first-generation BI analog, IGla100 (39–41). We speculate that this might be why IDeg was better than IGla100 but not superior to IGla300 on TIR. Since nearly half of the original articles did not report the types of IGla they used, we were concerned that the pooled results would be inappropriate due to potential heterogeneity. Fortunately, most of our meta-analysis results were non-heterogeneous, except for MODD in T1DM. In the pooled result of MODD, both T1DM and T2DM showed no difference between IDeg and IGla, but there was considerable heterogeneity in T1DM. To find out the cause of heterogeneity, we reviewed the baseline characteristics between studies and found no significant differences except for the duration of follow-up. The results of one study (18) with a 24-week follow-up differed significantly from the other study (27) with a 4-week follow-up. Therefore, we hypothesized that follow-up time might be one of the sources of heterogeneity. Interestingly, the study with a shorter follow-up time used IGla300, while the one with a longer follow-up did not report the type of IGla. We speculated that this might be another reason for the heterogeneity.

Our study showed that IDeg was superior to IGla in reducing fasting glucose fluctuations in T1DM and T2DM. However, we cannot ignore an important problem at the same time. Although studies have shown that IDeg had an advantage over IGla in controlling fasting glucose variability, studies comparing IDeg and IGla in fasting glucose variation are still insufficient, and existing studies only confirmed that IDeg was superior to IGla100. Three studies evaluating the glucose excursion of FBG did not report the type of IGla (17–19). Therefore, it is still unclear whether IDeg is superior to IGla300 in reducing the glucose variability of FBG. For various reasons, IDeg was superior to IGla in reducing FBG variability, which the following may explain. According to Heise et al., IDeg was four times lower than IGla in diurnal variation in total metabolic effect (42). When IDeg is injected subcutaneously, it forms soluble multimers that break down into monomers that are slowly and continuously absorbed into the circulation (43), which may make the pharmacodynamics of IDeg more stable when acting on humans. Additionally, Zinman et al. reported that day-to-day fasting glycemic variability was significantly associated with severe hypoglycemia (44) and it is well known that hypoglycemia is an essential manifestation of blood glucose fluctuation. Of note, previous studies showed that IDeg was associated with a statistically significantly lower rate of hypoglycemia in comparison with IGla (45–48). Besides, IDeg and IGla, ultra-long-acting basal insulins, tend to control fasting blood glucose rather than glucose throughout the day. Hence, IDeg could be more advantageous than IGla in FBG variability. For other metrics, including MAGE, mean of 24 h, CV of 24 h, SD of 24 h, AUC, and M-value, which needed to be measured throughout the day, the difference could not be so noticeable. A previous study pointed out that a common feature of many traditional markers of glucose variability (including MAGE, SD, and M-value) is their tendency to hyperglycemia. For purely numerical reasons, these metrics are primarily affected by hyperglycemia and are less sensitive to hypoglycemia (49). Therefore, we speculated that one of the reasons these indicators did not have statistical significance between IDeg and IGla might be that most studies assessed these metrics with a baseline HbA1c of <9%. Of course, more future studies are warranted to confirm this.

This study has two significant strengths. First, to the best of our knowledge, this is the first meta-analysis to assess the efficacy of glycemic variability between IDeg and IGla. Previous meta-analyses tended to focus on assessing the effect of lowering blood glucose and the incidence of hypoglycemia between the two insulins (20, 21), neglecting to evaluate the effect on glucose fluctuations systematically. Second, this study evaluated the effect of IDeg and IGla in reducing glucose variations from multiple perspectives, not just one; consequently, a comprehensive assessment was obtained. To some extent, the results of our study could have a certain reference value for the clinical application of basic insulin.

However, several limitations in our study should be acknowledged. First, the biggest limitation of this article was that nearly half of the included articles did not specify the type of IGla and 70% did not specify the type of IDeg, which greatly limited our analysis based on insulin preparation and insulin regimen. Second, not every study reported all indicators of blood glucose fluctuations; thus, although there were 14 studies, there were just several studies on each metric, which is why publication bias was not conducted. Third, the trials included in our study were undertaken mainly in China and Japan, which may affect the generalizability of the research. Fourth, most studies included a limited number of sample sizes in our research, except one trial with 498 cases evaluating the efficacy of TIR and one study with 7,637 cases assessing the efficacy of MBG, and the number of participants in other metrics of glucose variation is still insufficient. Fifth, most studies are crossover trials, and their mixing with parallel design may have potential bias, although we have tried our best to minimize the skew in the process of extracting data. Despite these limitations, we believe that vital information can be obtained from this research for further study.

In conclusion, IDeg was found to be superior to IGla in reducing fasting glucose variability in both T1DM and T2DM, but due to the limitations of the original study, it is still unclear whether IDeg is superior to both IGla100 and IGla300. Moreover, studies comparing the efficacy of IDeg and IGla in fasting glucose fluctuations are still needed. In T2DM, IDeg had a longer TIR than IGla100 but not longer than IGla300. For other indicators of blood glucose variation, including SD of 24 h, MAGE, MBG of 24 h, CV of 24 h, MODD, AUC, and M-value, no significant differences were identified between IDeg and IGla, regardless of T1DM or T2DM. As both IDeg and IGla300 are second-generation insulin analogs, their PD and PK properties are improved compared with first-generation insulin analogs. From the literature we searched, we discovered that studies comparing IDeg and IGla300 are still insufficient. Therefore, more well-designed randomized controlled trials comparing IDeg and IGla300 for different metrics of glucose fluctuations are still necessary.

## Data Availability Statement

The original contributions presented in the study are included in the article/supplementary material. Further inquiries can be directed to the corresponding author.

## Author Contributions

YY and QC contributed to the study conception and design. The collection and extraction of data were performed by YY and CL. Evaluation and analysis of data were performed by YY and TL. The first draft of the manuscript was written by YY. QC provided guidance and resolved disagreements in the process. All authors listed have made a substantial, direct, and intellectual contribution to the work and approved it for publication.

## Funding

This study was supported by the Sichuan Science and Technology Program (2019YFS0085). The funder was not involved in the study design, collection, analysis, interpretation of data, the writing of this article or the decision to submit it for publication.

## Conflict of Interest

The authors declare that the research was conducted in the absence of any commercial or financial relationships that could be construed as a potential conflict of interest.

## Publisher’s Note

All claims expressed in this article are solely those of the authors and do not necessarily represent those of their affiliated organizations, or those of the publisher, the editors and the reviewers. Any product that may be evaluated in this article, or claim that may be made by its manufacturer, is not guaranteed or endorsed by the publisher.
